# The concept for hard X-ray beamline optics at SLS 2.0

**DOI:** 10.1107/S1600577524003163

**Published:** 2024-05-31

**Authors:** Benedikt Roesner, Joerg Raabe, Philip R. Willmott, Uwe Flechsig

**Affiliations:** a Paul Scherrer Institute Forschungsstrasse 111 5232Villigen PSI Switzerland; Paul Scherrer Institute, Switzerland

**Keywords:** SLS 2.0, hard X-rays, monochromators, optical design

## Abstract

In the scope of the latest upgrade of the Swiss Light Source, five hard X-ray beamlines will be constructed or rebuilt. To use synergies between these beamline projects, a concept is developed here for hard X-ray beamlines that is tailored to the new storage ring. Herein, this concept is described from the source, via the front end, to the beamline optics. The latter will be outlined in detail, including a new and modular concept for hard X-ray monochromators, focusing optics and heat-load management. With a simple, easy-to-operate and robust beamline design, the new beamlines will greatly profit from the increased brilliance of the new storage ring. The performance increase is up to four orders of magnitude, while the beamline concept allows for the broad application of experimental techniques, from propagation-based methods, such as phase-contrast tomography, to imaging techniques with nanometre resolution. At the same time, spectroscopy experiments are possible as well as high-performance serial X-ray crystallography.

## Abstract 2

A simple, easy-to-operate and robust beamline design is presented for five new hard X-ray beamlines in the scope of the latest upgrade of the Swiss Light Source. The performance increase is up to four orders of magnitude with respect to the current storage ring and beamline design.

## 1. Introduction

The Swiss Light Source (SLS) currently hosts 17 beamlines, of which eight offer a variety of techniques in the hard X-ray regime. The TOMCAT beamline (Stampanoni *et al.*, 2006 ▸) is dedicated to tomographic microscopy between 8 and 45 keV; the three crystallography beamlines PX I, PX II and PX III offer protein crystallography between 5.5 and 20 keV; the Materials Science beamline (Willmott *et al.*, 2013 ▸) uses diffraction methods between 5 and 38 keV, the cSAXS beamline provides ptychography (Holler *et al.*, 2017 ▸) and small-angle scattering (Bunk *et al.*, 2009 ▸) between 4.4 and 18 keV; and the SuperXAS (Müller *et al.*, 2016 ▸) and MicroXAS (Borca *et al.*, 2009 ▸) beamlines are dedicated to spectroscopic techniques between 3 and 35 keV.

The upgrade of the SLS to SLS 2.0 will include an increase in the storage-ring energy from 2.4 to 2.7 GeV, and a significantly lower horizontal emittance (Streun *et al.*, 2018 ▸; Willmott *et al.*, 2021 ▸) as a result of replacing the triple-bend achromats with seven-bend achromats. Accordingly, the hard X-ray beamlines will gain a huge factor in performance (brilliance as well as usable flux) compared with that at the present storage ring, due to not only the reduced emittance by approximately a factor of 37 but also innovations in source technologies, especially regarding undulators and superbends. In addition to the existing beamlines, two new beamlines are currently under construction: the I-TOMCAT beamline is going to take advantage of a new insertion device (ID) based on a high-temperature superconductor and will offer a complementary set of methods to the already existing TOMCAT beamline, which will be equipped with a new superbend magnet with 5 T (compared with the current 2.9 T source) and will be renamed S-TOMCAT, while the new Debye beamline will offer a similar experimental palette as the SuperXAS beamline to an industrial–academic user consortium. After the upgrade, SLS 2.0 will thus host ten hard X-ray beamlines for user operation.

To strengthen the hard X-ray capabilities of the upgraded synchrotron, four of the existing ID beamlines – PX I, PX II, cSAXS and MicroXAS – as well as the PX III superbend magnet beamline will be completely rebuilt. These ID beamlines, as well as the new I-TOMCAT beamline, will be equipped with new IDs, revised front ends and state-of-the-art optical elements tailored to the upgraded storage-ring specifications. The quite similar fundamental concept of these beamlines allows us to take advantage of strong synergies in designing the undulators, the front-end components and the optical elements. Regarding the latter, we were able to develop a unified and modular concept that can be integrated into the optical design of all five hard X-ray ID beamlines mentioned above. In this publication, we present this new concept. We will explain the overall approach, and important aspects for beamline design related to the sources and front ends. Furthermore, we will describe the new unified monochromator concept, including aspects of stability, expected performance, power management and radiation safety. The different operation possibilities, focusing capabilities and expected performances are addressed at the end of this article.

## 2. Concept for hard X-ray beamline optics

### 2.1. Overall design

In order to equip five hard X-ray beamlines at the undulator sources of the SLS 2.0 with new optics, we have chosen an approach that provides the advantages of synergies, while maintaining the flexibility to provide the individual beamlines with tailored optical elements that optimally fulfil the needs of the planned experiments. The development of the concept for our hard X-ray beamlines was driven by the idea of providing a design that offers high performance while still being simple, robust, and having commonalities regarding maintenance and repair. Keeping this in mind, we have started from the proven successful design of the existing hard X-ray beamlines, in which the undulator radiation is first directed onto a crystal monochromator, followed by subsequent focusing optics.

**Figure 1:**
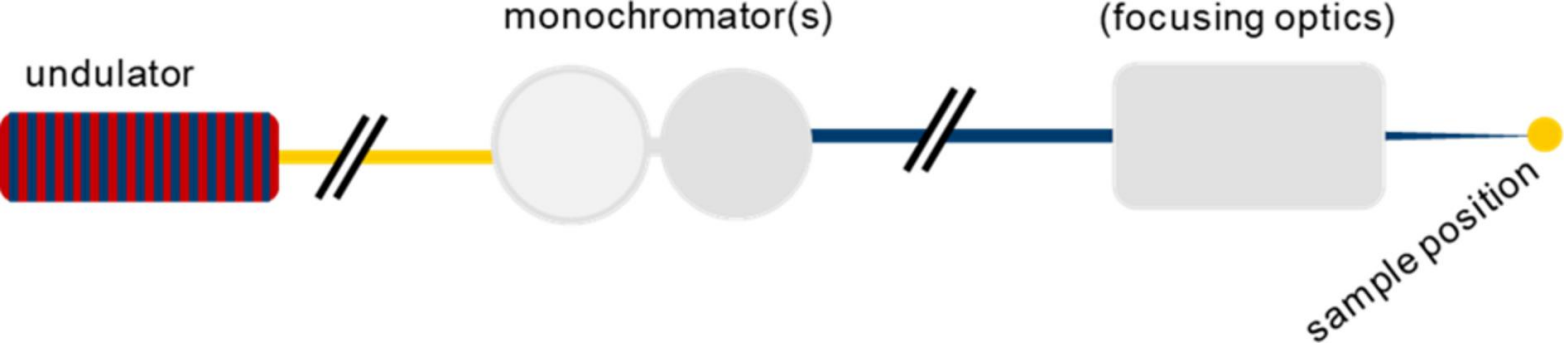
A schematic illustration of the design for hard X-ray ID beamlines at SLS 2.0 (a top view). After the undulator and front end, the beamline is composed of a section that accommodates one or two monochromators with the same horizontal beam offset, with only one to be used at any one time. Subsequently, different kinds of focusing optics can be installed, such as a KB system or Fresnel zone plates. The focusing optics can also be omitted to use an unfocused beam for propagation-based imaging applications.

The core part of this concept is a simple and modular building block containing one or two state-of-the-art hard X-ray monochromators that are illuminated directly by the undulator radiation and can be placed in the beam individually, *i.e.* not in sequence. This building block also includes the power-management and radiation-safety concepts, and can be tailored to the needs of each beamline. Downstream of the monochromators, we profit from the flexibility to install different kinds of focusing optics (Fig. 1 ▸). Our standard solution for this task is the use of a Kirkpatrick–Baez (KB) mirror system with variable focal length. Alternatives to KB mirrors are Fresnel zone plate lenses, compound refractive lenses (CRLs), a combination of those, or the omission of focusing optics, notably in the propagation-based tomographic experiments carried out at I-TOMCAT.

### 2.2. Source and front ends

At the current SLS, in-vacuum undulators with 19 mm period generate hard X-rays between 5 and 30 keV. Their gap size ranges between 4.5 and 7 mm in operation, and can be opened to more than 20 mm. For the new machine parameters of SLS 2.0, a new generation of undulators will be installed. These devices have a modular design and can be equipped with up to 185 magnet pole pairs with a periodicity between 16 and 18 mm (Braun *et al.*, 2021 ▸). The gap range is between 4.0 and 13.4 mm, providing *K* values up to 2.0. The standard design, which will be used for PX I, PX II and cSAXS, is a U17 undulator (3 m in length, λ = 16.8 mm, *N* = 176). The reduced horizontal emittance and the increased length of the radiation source lead to a significant increase in brilliance by well over two orders of magnitude. The same effects also lead to a significantly smaller horizontal divergence of the photon beam, which enables us to limit the beamline acceptance using adjustable slits. As a result, the power load that is dissipated into the beamline will be smaller than in the original SLS. For this reason, the front-end group has developed a new slit design to be used in the ID front ends as well as downstream at the beamlines.

### 2.3. Monochromator concept

One of the key considerations in beamline design planning is the configuration of monochromators. Currently, the hard X-ray monochromators of the SLS have a vertical offset of typically 50 mm. In addition, the current design includes a sagittal bender (Schulze-Briese *et al.*, 1998 ▸), which bends the second crystal in the sagittal direction with respect to the beam to obtain horizontal focusing. This specific geometry means that the focusing, *i.e.* the sagittal bending radius, varies with the photon energy, and as such poses limitations in beam stability, as well as in accuracy and reproducibility of the focusing conditions.

For the new monochromator concept, we evaluated two major questions: firstly, whether to adopt a horizontal or vertical configuration; and secondly, whether to operate two monochromators – a multilayer monochromator and a crystal monochromator – sequentially or individually. New monochromator designs have recently become popular that combine a multilayer monochromator and a removable double channel-cut crystal monochromator downstream of the multilayer monochromator in sequence (Katayama *et al.*, 2019 ▸; Yabashi & Tanaka, 2017 ▸). One advantage of these schemes is that the power load can be partially dissipated on the broadband (multilayer) monochromator and, downstream of this, also on the narrowband (crystal) monochromator. If an upstream reflecting mirror to cut off the ID’s higher harmonics is included, it is possible to distribute the power even more for sophisticated thermal management. In addition, the higher harmonics of a multilayer monochromator are non-integer multiples of its fundamental, so that the higher harmonics of the subsequent crystal monochromator are also suppressed efficiently.

By calculating the thermal load that enters the beamline with *SPECTRA* (v. 11.0) (Tanaka & Kitamura, 2001 ▸), we find, however, that the effect of this arrangement seems not to pay off. For the full front-end opening, we expect a power load below 400 W, which is reduced by an 80 µm-thick diamond window to 290 W. A platinum-coated mirror with 3 mrad incidence angle would absorb less than 50 W, still leaving 240 W incident on a multilayer monochromator. A chosen undulator harmonic that passes the monochromator would still contain a power load that can reach 10 W, which means that a subsequent monochromator still would have to be cooled actively. If the front-end slits are used to limit the acceptance to, for instance, typically four sigma of the beam divergence, the power-absorbing effect of an additional mirror shrinks even more. In this case, the total beam energy is reduced from ∼170 to ∼120 W by the vacuum window, and finally to ∼100 W by the mirror. In summary, the heat-load reduction of an upstream X-ray mirror would only be 20–50 W. We should note at this stage that the integrated power load at the original SLS is almost twice as large under similar conditions (300–400 W at a typical cryocooled monochromator), whereas the (on axis) power density will triple at SLS 2.0. Finite element analysis has shown that the reduced power load leads to a strong reduction in local heating of the monochromator crystals, despite the strongly increased power density, due to the heat transfer to the cooling system being the limiting factor. Keeping this in mind, the advantages of integrating an additional optical element to reduce the heat load must be weighed against the additional complexity, risk and costs of this strategy. We have therefore decided to keep the monochromator as the first optical element. An exception is the MicroXAS beamline, which will take advantage of two pre-mirrors to control the focusing conditions and the divergence upstream of the monochromator for possible nano­focusing and high-resolution spectroscopy.

Regarding the choice of vertical or horizontal dispersion geometry, the lower emittance of the upgraded synchrotron lattice permits a horizontal dispersion geometry, since the divergence is much smaller, thus not limiting the energy resolution. As the photon-energy range of most of the new beamlines will start at 5–6 keV, the lower reflectivity caused by polarization effects will not have a significant impact on the reflectivity of the crystals. We thus carefully considered the horizontal dispersion geometry, which offers the advantage of having the centre of gravity on top of the Bragg axis. This geometry was, for instance, realized in the BioNanoprobe beamline at the Advanced Photon Source (Lai *et al.*, 2007 ▸) or in beamline I13 at the Diamond Light Source (Wagner *et al.*, 2011 ▸, 2013 ▸), or, more recently, at several beamlines around the world. During a technical survey before deciding on the monochromator concept, the authors found that state-of-the-art horizontally dispersing monochromators outperform the mostly older vertically dispersing models in terms of vibration stability. Although it is unclear how much of the observed stability increase is due to the horizontal geometry and how much is contributable to the more compact design and smaller beam offsets of such monochromators, the vibration stability of compact horizontal crystal monochromators has been demonstrated to be as low as 25 nrad r.m.s. under liquid-nitro­gen-cooling conditions, as determined by the cumulative spectral density in the frequency domain between 1 and 2500 Hz (Kristiansen *et al.*, 2016 ▸). We thus decided to implement a horizontal geometry for the new monochromators.

**Figure 2:**
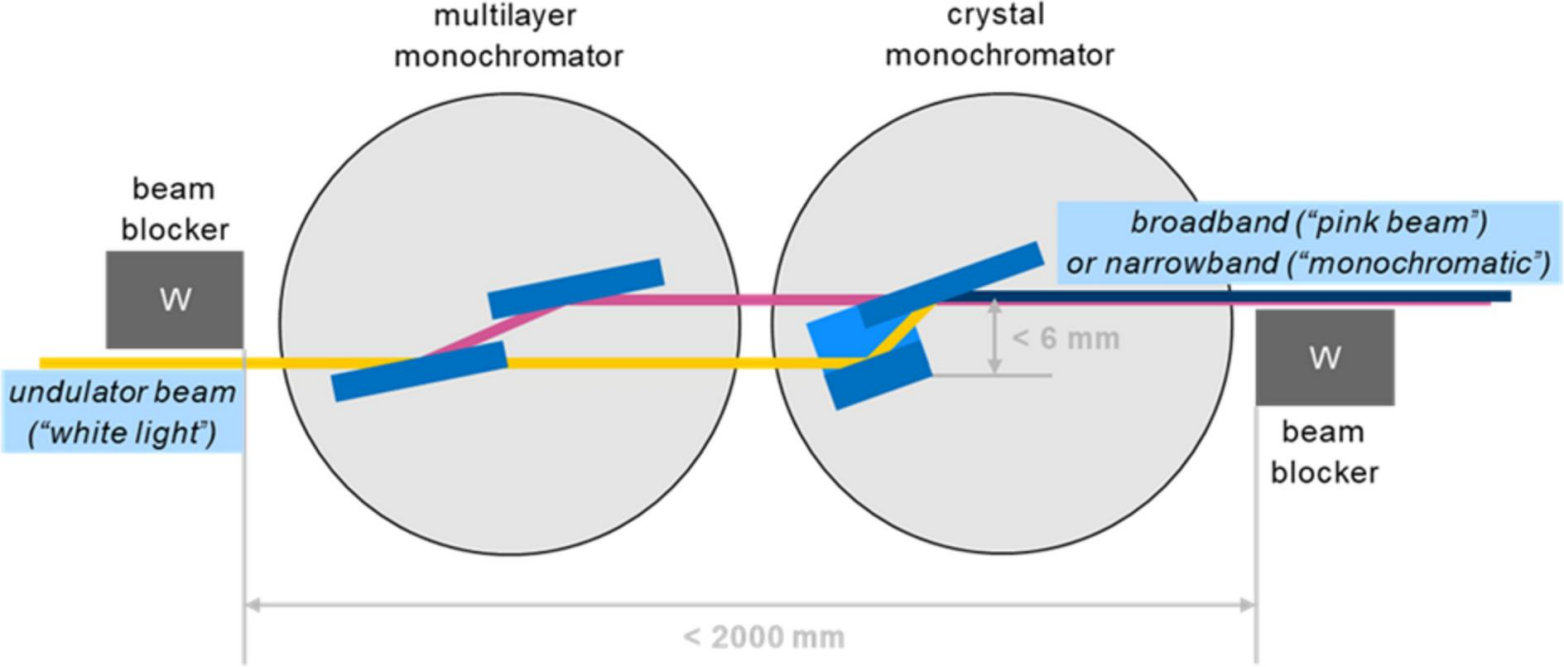
A top view of the monochromator concept for hard X-ray ID beamlines at SLS 2.0. The concept foresees a modular building block that can accommodate one or two independent monochromators. Alternatively, one monochromator can be omitted, or integrated in the design later. The monochromators will have a maximum beam offset of 6 mm. As the first optical surface is placed within the direct undulator beam, the monochromators will be cryocooled. Two tungsten beam stoppers prevent bremsstrahlung from leaking into the experimental hutch and guarantee radiation safety.

To satisfy the needs for all the newly built beamlines in terms of monochromator choice and geometry, our new monochromator concept is designed as a simple and modular building block that can contain two state-of-the-art hard X-ray monochromators: a multilayer monochromator and a crystal monochromator. The monochromators are directly illuminated by the undulator radiation and can be inserted in the beam individually, *i.e.* not in sequence. Both monochromators are cooled with one cryocooler, so that the conditions are stable, facilitating a rapid exchange from one to the other. The beam offset is fixed at 6 mm for the multilayer monochromator and is the maximum value for the crystal monochromators at their highest deliverable photon energy. This has been evaluated as the ideal compromise between a reasonable acceptance of the multilayer monochromator and the minimum offset required by the radiation-safety concept. In this way, both monochromators are independent in terms of installation, operation and beam stability. Upstream and downstream of the set of the two monochromators, tungsten beam blockers are installed (Fig. 2 ▸). It is also possible to bring the beam blockers closer together and to install one monochromator only.

The new monochromators are designed with the major focus on three aspects that have been deemed most important: simplicity, robustness and stability. The latter, especially, is currently a limiting factor at the SLS beamlines, due mainly to the relatively large beam offsets that are used. Consequently, we have decided to follow the recent development for hard X-ray monochromators and install horizontal offset monochromators using either a pseudo channel-cut geometry or one where only the gap size is adjustable, but not the positions of the optical elements along the beam. This also means accepting a moving footprint on at least one of the optical elements within each monochromator. This strategy allows one to minimize the number of motions. Together with a small beam offset (see above), a high beam stability is anticipated. High-quality optics will ensure the necessary beam quality for techniques based on lensless imaging and diffraction.

#### 2.3.1. Double multilayer monochromators

Multilayer optics offer the fundamental advantage that they provide broadband synchrotron radiation with bandwidths that are typically in the range of 1–3% of the chosen photon energy. This is particularly interesting for applications that profit from high flux, such as (high throughput) tomographic imaging, ptychography and some diffraction methods. At SLS 2.0, multilayer monochromators will be installed at the I-TOMCAT beamline, at the cSAXS beamline and at the PX I beamline. The other crystallography beamline, PX II, and the MicroXAS beamline have space reserved for a multilayer monochromator to be added later.

The monochromators at I-TOMCAT and cSAXS will be equipped with multilayer mirrors that accommodate two or three different coatings. These optical elements will be optimized to provide high flux. Table 1 ▸ summarizes the parameters planned for the I-TOMCAT beamline. The resulting properties for reflectivity have been measured on the multilayer-coated substrates that were recently installed at the twin beamline of I-TOMCAT, S-TOMCAT.

**Figure 3:**
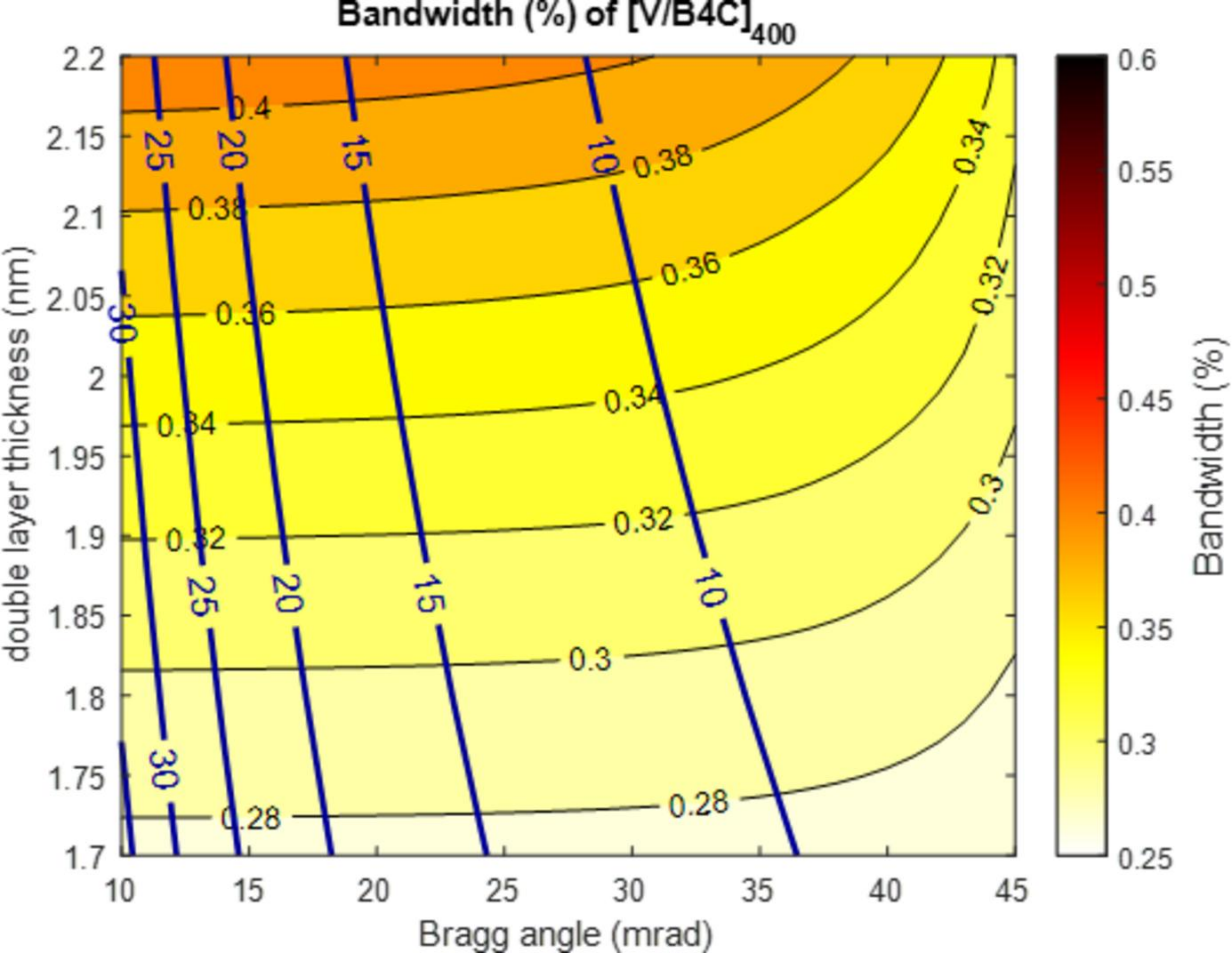
Expected bandwidth of a [V/B_4_C]_400_ multilayer system with Γ = 0.3 and 3 Å r.m.s. surface roughness. The plot shows the expected bandwidth as a function of Bragg angle (incidence angle) and double-layer thickness. The dark blue lines represent the corresponding photon energy in keV.

While the I-TOMCAT and cSAXS beamlines will be optimized for high flux, PX I aims at operation with low and medium bandwidth. For this purpose, specific multilayer combinations will be used that are like those utilized at ID29 at the ESRF and at MicroMAX at MAX IV. In particular, the low-bandwidth stripe on the multilayer mirrors will be M/B_4_C, where M is a metal layer of titanium or vanadium. Fig. 3 ▸ shows the calculation of the bandwidth of a [V/B_4_C]_400_ multilayer system^1^ with a gamma value of 0.3, including a substrate roughness of 1 Å and a layer roughness of 3 Å. The calculations were carried out with the *REFLEC* (Schäfers & Krumrey, 1996 ▸) code. The chosen multilayer spacing, *d*, will be 2.0 nm. Similar multilayers have been demonstrated to provide energy resolutions of 0.34% at the ESRF (Morawe, 2019 ▸). Thermal analysis has shown that the monochromator for PX I must be cryocooled to avoid a heat bump that would otherwise shift the rocking curve so much that the reflectivity would drop by a factor of two.

**Figure 4:**
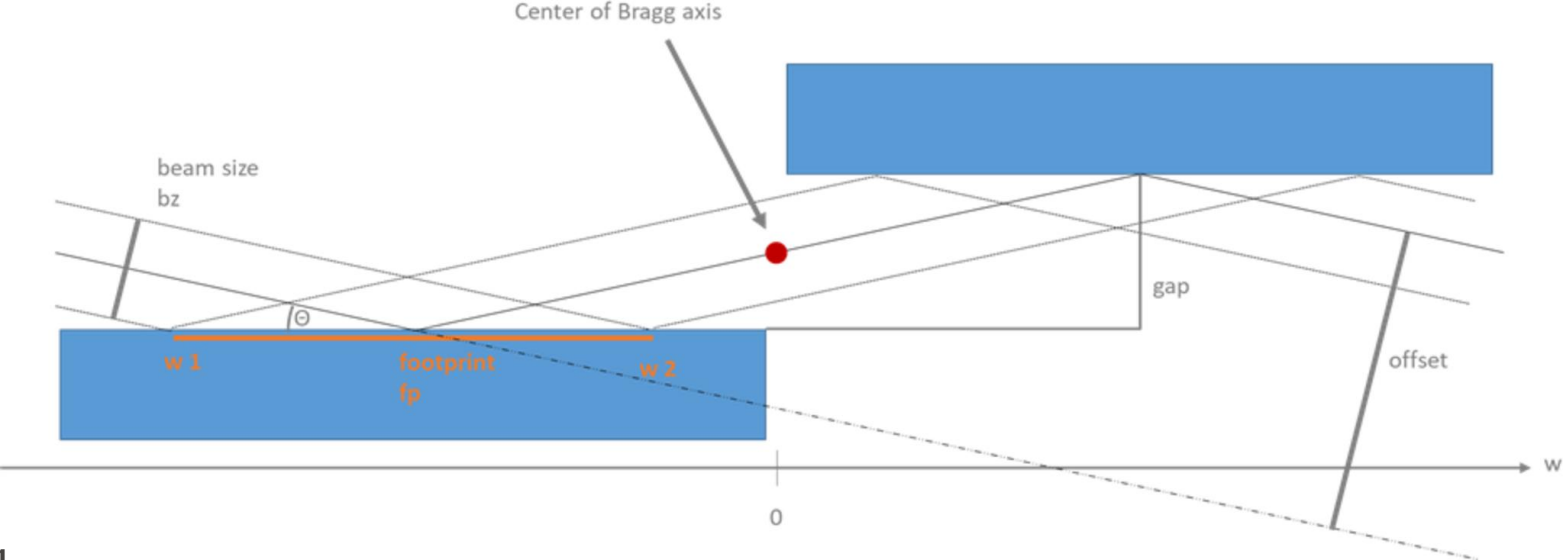
The geometry of the multilayer monochromators. The rotation axis is in the middle of the gap between the two optical elements.

The geometry of the monochromators has been chosen to be centrosymmetric with the rotation axis in the middle (see Fig. 4 ▸). In this way, we expect that the weight of the multilayer mirror assembly will not distort the overall geometry, especially due to the horizontal deflection arrangement. The angular range of the monochromators will be 10–35 mrad (incident angle, I-TOMCAT starting at 5 mrad). The length of the optical elements is 270 mm (cSAXS and PX I) or 345 mm (I-TOMCAT).

#### 2.3.2. Channel-cut monochromator

Channel-cut monochromators are known for their mechanical stability and robustness. The intrinsic feature of using a single crystal as the optical element instead of two individual crystals means that the sensitivity of a channel-cut crystal to mechanical vibrations and thermal fluctuations is much lower than in a double-crystal monochromator. Furthermore, channel-cut monochromators allow one to minimize the number of motions to the Bragg axis only (ignoring the lateral motion to remove the monochromator from the beam). Overall, this design is perfectly suited to obtain the highest beam stability for the cSAXS beamline.

**Figure 5:**
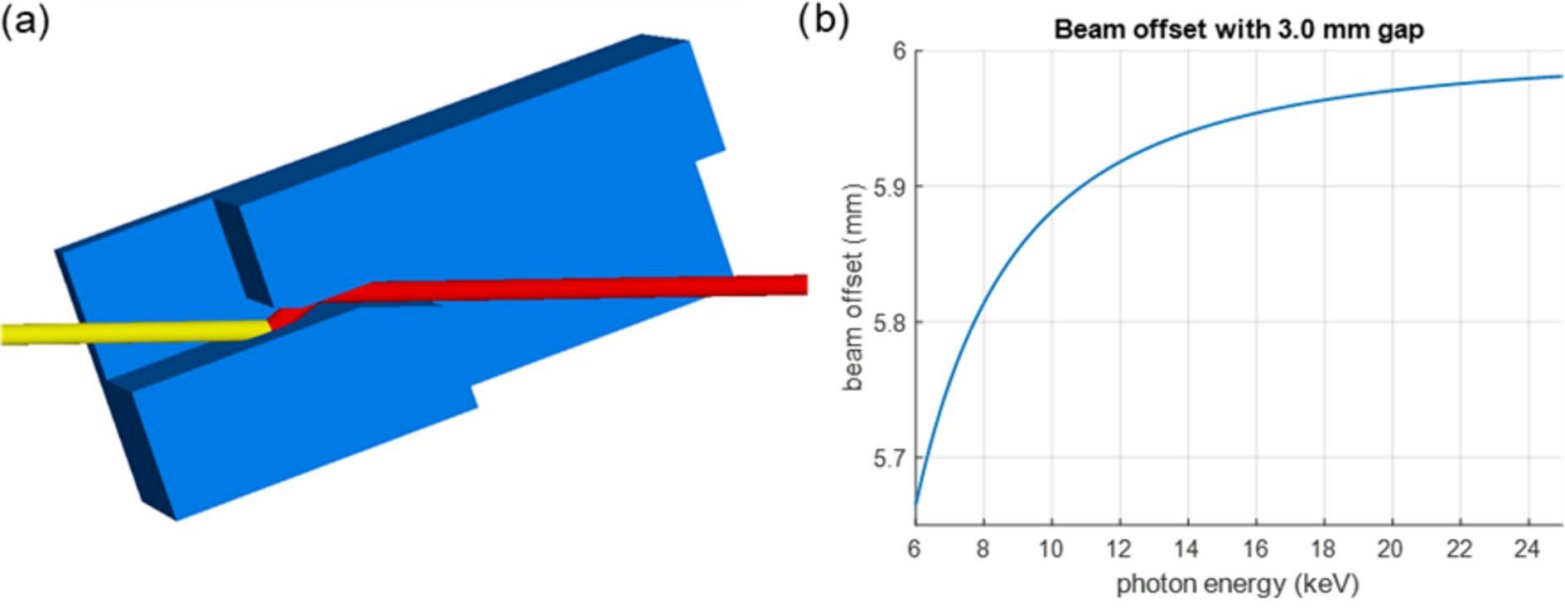
(*a*) A model of the channel-cut crystal for cSAXS at 6 keV (a top view). The edges of the optical surfaces are chamfered to avoid clipping the beam at high angles. (*b*) Beam offset for an Si(111) channel-cut crystal with a 3 mm gap.

The primary disadvantage of channel-cut monochromators is the changing beam offset when changing the photon energy. Nonetheless, the chosen small 3 mm channel width of the Si(111) crystal results in a drift of the offset that is acceptable. Strategies to minimize the offset variation, such as asymmetric polishing (Thompson *et al.*, 2004 ▸), have been evaluated but will not be pursued at the moment. For the full energy range from 6 to 25 keV as defined for the cSAXS beamline, the offset change is ∼320 µm, resulting in a beam offset between 5.63 and almost 6.0 mm (see Fig. 5 ▸).

#### 2.3.3. Double-crystal monochromators

In contrast to the cSAXS beamline, the PX I and PX II beamlines require a fixed beam offset because of an exactly defined sample interaction point that must stay fixed when the energy is changed or scanned. These beamlines will therefore be equipped with a double-crystal monochromator with gap adjustment. Piezo-driven fine pitch and fine roll stages ensure precise control of the beam position that can use a feedback signal from a beam monitor.

#### 2.3.4. Front-end power management

**Figure 6:**
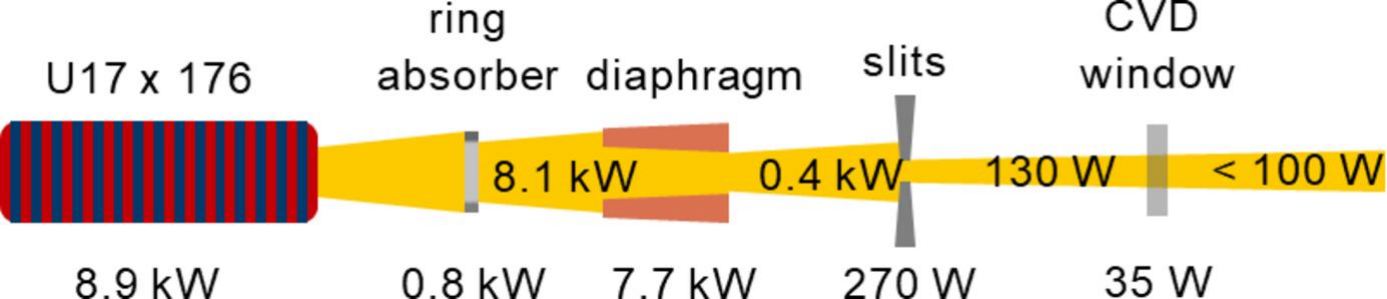
Power management along the front end of the beamline for the minimum gap (4.0 mm) at a *K* value of 2.0. A major part of the power is dissipated in the front end, which contains a ring absorber, a diaphragm, a set of high-power slits and a diamond window. The power that is transmitted into the beamline is less than 100 W.

A crucial point for the new beamline concept is the power management. As the new undulators are more powerful than the current IDs, the power that is dissipated into the beamline increases significantly. The maximum power of the old IDs (5.0 kW) will almost double to 8.9 kW. This amount of power must be managed efficiently to avoid the power load on the optical components being too high. On the other hand, the smaller horizontal emittance of the machine allows for smaller horizontal acceptance angles compared with the original SLS, so that most of this power can be dissipated upstream of the monochromator in the beamlines’ front ends. Fig. 6 ▸ shows the power management along the front end. A ring absorber follows the undulator, which takes up to 0.8 kW. The major power load is absorbed by a specifically designed diaphragm, which will dissipate up to 7.7 kW of heat. The remaining power of 400 W is taken up by the beamline slits, which absorb 270 W at 5σ beam acceptance, and a vacuum window that absorbs up to another 35 W. The remaining beam thus has a power of less than 100 W under operational conditions, which is significantly lower than the previous typical absorbed power of 300–400 W. For fully open front-end slits, we expect up to 350 W in the beamline.

Although the total power load in the beamlines is moderate, the beam’s low divergence associated with the upgrade means that the monochromators must still be cooled with liquid nitro­gen. The perpendicular on-axis power density can be as much as 210 W mm^−1^ at the monochromator positions, which leads to a projected power density of up to 64 W mm^−1^ on the tilted optical elements. Cryocooling ensures that the thermal expansion coefficient of the optical elements is close to its zero crossing at 123 K (Swenson, 1983 ▸). To keep vibrations induced through the cryogenic nitro­gen at a minimum, the cooling circuits are designed to run as smoothly as possible, which is achieved by working at higher pressures than currently used (5–10 bar working pressure instead of 2–3 bar) and by reducing the speed of the cryopump as much as possible to values below 30 Hz, ideally targeting <21 Hz. This strategy is possible due to a higher boiling point of liquid nitro­gen at higher pressures so that we can work with a reduced flow compared with lower pressures.

### 2.4. Focusing optics

#### 2.4.1. Kirkpatrick–Baez mirrors

**Figure 7:**
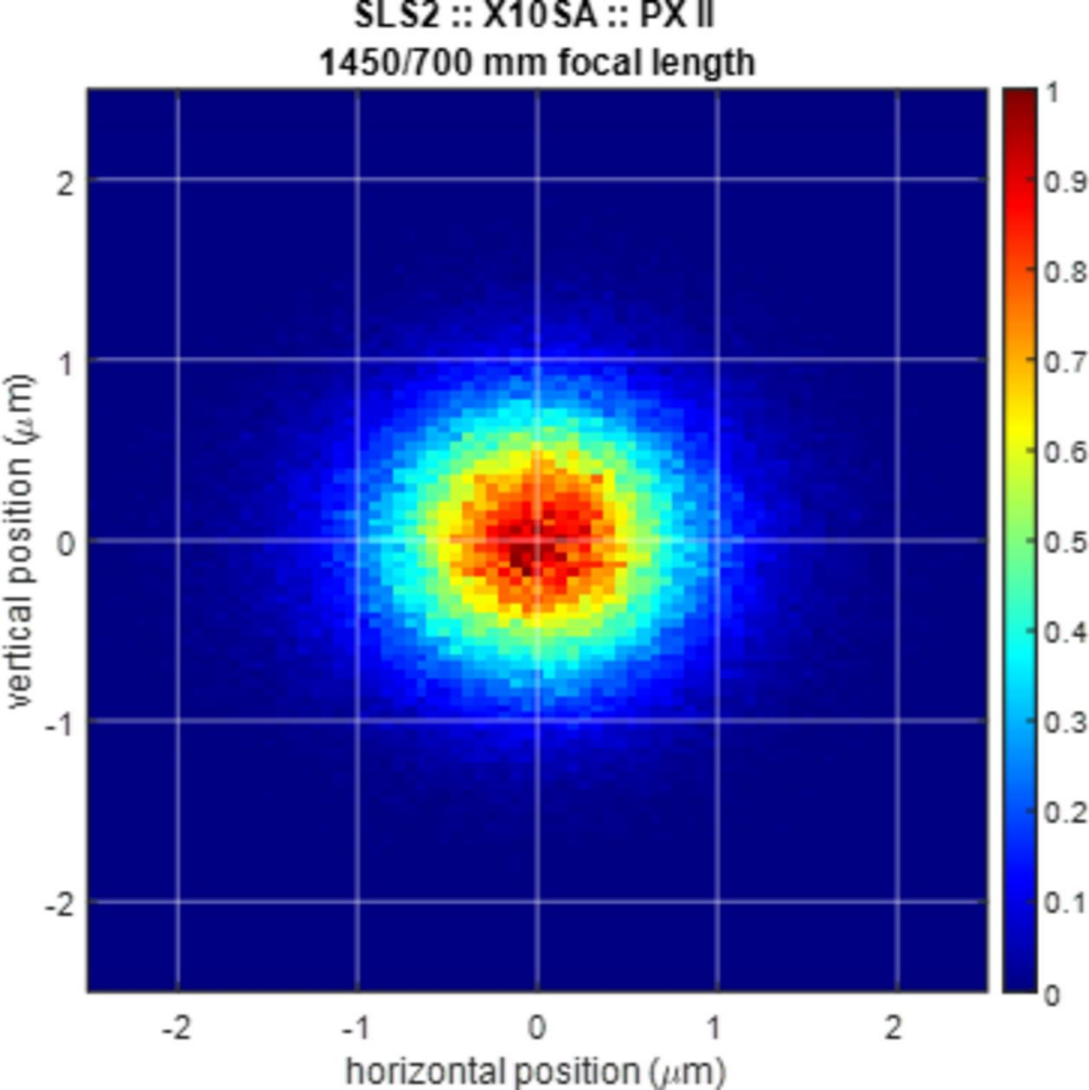
Ray-tracing result showing the focus of the PX II beamline at 12.4 keV. The slope error is assumed to be as low as 0.1 µrad r.m.s. The colour bar shows the normalized beam intensity.

The cSAXS, PX I and PX II beamlines will be equipped with three bendable KB mirror systems (Kirkpatrick & Baez, 1948 ▸) to achieve a good and reproducible focus. The design of these mirror systems is based on the design of the KB systems that are installed at the Aramis beamlines at SwissFEL (Juranic *et al.*, 2019 ▸; Ingold *et al.*, 2019 ▸). The mirrors are generally arranged with the vertically focusing mirror on the upstream side, followed by the horizontally focusing mirror. With focal lengths of 700 mm (PX II), 2000 mm (PX I) and 3.0 to 10 m (cSAXS) from the horizontally focusing mirror, and incidence angles between 2 and 3 mrad, the expected focus sizes range from 1.1 µm × 1.4 µm to 28 µm × 25 µm. Fig. 7 ▸ shows a ray-tracing result obtained with the ray tracer *PHASE* (Bahrdt & Flechsig, 1997 ▸; Bahrdt *et al.*, 2014 ▸) for the tightest focus. The focusing capabilities with short focal distances are limited by the figure error of the mirror rather than the fundamental Abbe limit. For this reason, the strongly bent horizontally focusing mirror at PX II has been designed with a barrel shape to achieve an almost perfect plane elliptical figure (Juanhuix *et al.*, 2019 ▸). Additionally, the mirrors are specified with slope errors below 100 nrad r.m.s. (cumulative spectral density for sizes between 1 mm and the length of the optical useful area). Finally, the vertically focusing mirrors are coated with multiple stripes: platinum, rhodium and an uncoated area, which can be changed for harmonic rejection if required.

The KB system for the cSAXS beamline is a special design challenge. Due to the wealth of scientific applications that require a clean and stable wavefront, the mirrors are designed with a minimum figure error in several configurations. The focusing modes range from the use of an unfocused beam that illuminates a set of Fresnel zone plates, with dynamic focusing onto a detector between 4 and 10 m downstream of the mirror system, to a focus on the sample position 3 m from the horizontally focusing mirror. To ensure high reproducibility, we have chosen a slightly convex mirror shape that is dynamically bent to a flat surface or to a concave focusing configuration. For the use of an unfocused beam, the vertically focusing mirror is completely removed.

#### 2.4.2. Diffractive focusing optics

Some applications require either a larger focus or a nanometre-sized focal spot. Whereas the spot size of a mirror system is limited by its geometry, meaning the focal distance and the size of the mirror system, diffractive optics are more flexible. Fresnel zone plates are widely used at the SLS.

For example, zone plates serve multiple purposes at the cSAXS beamline. Its standard zone plate has a diameter of 120 µm and an outermost zone width of 60 nm. The zone plates are typically made of gold and are 1.0–1.2 µm thick. With these parameters, the zone plates are particularly suited for a photon-energy range between 6 and 12 keV, where they can be used with focal lengths between 35 and 70 mm.

The simplest use case is to create a nanometre-sized focus with spot sizes below 100 nm. More recent developments comprise the use of Fresnel zone plates that induce artificial wavefront errors (distorted zone plates) (Odstrčil *et al.*, 2019 ▸) or imprint phase vortices (spiral zone plates) (Vila-Comamala *et al.*, 2014 ▸; Ribič *et al.*, 2017 ▸; De Ninno *et al.*, 2020 ▸; Fanciulli *et al.*, 2022 ▸; Wätzel *et al.*, 2022 ▸). In order to use zone-plate optics, the KB mirror system of the beamline and the following elements are designed in a way that allow operation with a flat horizontal-deflection mirror for harmonic rejection only. At I-TOMCAT, the use of an axicon is foreseen for focusing purposes (Willmott *et al.*, 2021 ▸).

#### 2.4.3. Other focusing schemes

Besides KB mirrors and Fresnel zone plate lenses, other methods can be implemented to focus X-rays. Some examples include the use of CRLs (Smither *et al.*, 1997 ▸; Snigirev *et al.*, 1998 ▸), polycapillary optics (MacDonald, 2010 ▸) or the omission of all focusing optics. The optical concept of the hard X-ray beamlines leaves room for the implementation of such solutions. For instance, the I-TOMCAT beamline has the option to work with an unfocused beam, or PX II will host a set of CRLs to be able to defocus the beam.

## 3. Beamline overview and performance increase

Of the ten hard X-ray beamlines at SLS 2.0, five beamlines are going to be upgraded and will take advantage of optical schemes that are based on the concept presented in this article: the I-TOMCAT beamline, the crystallography beamlines PX I and PX II, the cSAXS beamline, and the MicroXAS beamline. Table 2 ▸ summarizes the beamlines and the major design choices.

**Figure 8:**
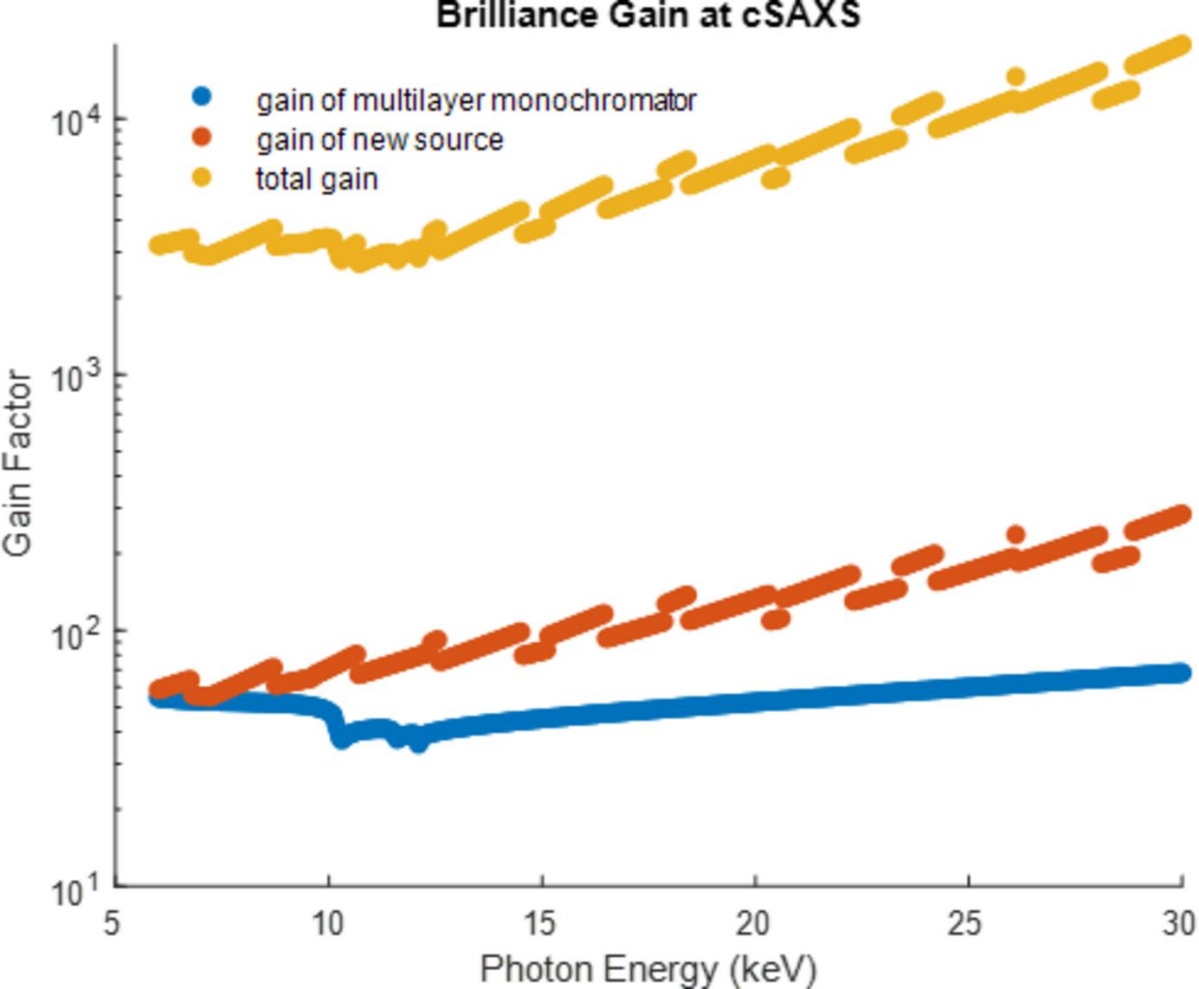
Performance gain of the cSAXS beamline after the SLS 2.0 upgrade and the installation of new optics. The contribution from using a multilayer monochromator instead of a crystal monochromator is shown in blue. The contribution of the ring upgrade in combination with the new ID is shown in red. The combined total gain factor of the beamline over the usable energy range is shown in yellow. However, the platinum-coated mirrors lead to a cut-off in beamline performance between 25 and 30 keV photon energy, which is not included in this plot.

Except for MicroXAS, these upgrades will be implemented during the main upgrade of the synchrotron that started in late 2023 and will be finished in spring 2025. Together with the upgrade of the storage ring, a huge leap in performance is expected. For instance, the cSAXS beamline is going to profit from several factors: the lower emittance and the new ID will provide a gain in brilliance with a factor between 60 and 300, dependent on the chosen photon energy; and the installation of the broadband monochromator will provide an increase of almost another two orders of magnitude. Overall, the performance increase of the beamline is going to be between 10^3^ and 10^4^ (see Fig. 8 ▸). In addition, the stability of the beamline is expected to increase significantly. These factors have the potential to enable faster experiments and increase the beamline’s resolution, allowing for higher resolving power, and rendering weak-contrast measurements, *e.g.* on magnetic samples, more feasible. Moreover, it will increase the throughput of samples to a degree that ptychography can contribute in statistically significant ways to demanding comparative studies (Willmott *et al.*, 2021 ▸). This shows that the high effort put into the beamline upgrades is more than justified and will lead to exciting new experimental vistas at the SLS 2.0.

## Figures and Tables

**Table 1:** Parameters for the monochromator stripes The parameter *d* is the spacing of a double layer, and Γ is the thickness ratio between the metal (reflecting) layer and the layer spacing (*i.e.* the thicknesses of the reflecting and transparent layers). The values for reflectivity and bandwidth have been measured at 8.0 keV on the multilayer-coated substrates that were installed at S-TOMCAT.

Stripe	Material combination	Number of double layers	*d* (nm)	Γ	Energy (keV)	Reflectivity (8 keV) (%)	Bandwidth (%)
# 1	Ru/C	100	4.0	0.32	4.4–30	82.3	3.0
# 2	W/B_4_C	130	3.0	0.33	5.9–40	81.3	2.1
# 3	W/B_4_C	200	2.2	0.33	8.0–55	74.3	1.1

**Table 2:** An overview of the hard X-ray beamline upgrades at the SLS

				Focusing scheme	
Beamline	Source	Broadband monochromator	Narrowband monochromator	Standard	Optional
I-TOMCAT	U15 (2025)	Double multilayer monochromator, 3 stripes	None	None	Axicon, Fresnel zone plate lenses
	U10 (2026)				
PX I	U17	Double multilayer monochromator, 2 stripes	Double-crystal monochromator	KB system, 2 m focal length	CRLs
PX II	U17	Optional at a later stage	Double-crystal monochromator	KB system, 700 mm focal length	CRLs
cSAXS	U17	Double multilayer monochromator, 2 stripes	Channel-cut monochromator	KB system, 3–10 m focal length	Fresnel zone plate lenses
MicroXAS	U16	Double multilayer monochromator	Double-crystal monochromator	Pre-focusing mirrors and KB system	Nanofocus KB system, Fresnel zone plate lenses

## Footnotes

1 [V/B_4_C]_400_ refers to 400 layer pairs that each consist of one metal layer (V) and one lighter spacer layer (B_4_C).
